# Performance of late pregnancy biometry for gestational age dating in low-income and middle-income countries: a prospective, multicountry, population-based cohort study from the WHO Alliance for Maternal and Newborn Health Improvement (AMANHI) Study Group

**DOI:** 10.1016/S2214-109X(20)30034-6

**Published:** 2020-03-18

**Authors:** The WHO Alliance for Maternal and Newborn Health Improvement Late Pregnancy Dating Study Group, Saikat Deb, Mohammad Said Mohammed, Usha Dhingra, Arup Dutta, Syed Mohammed Ali, Pratibha Dixit, Mohammed Hamad Juma, Massoud Juma Hassan, Sunil Sazawal, Imran Nisar, Muhammad Ilyas, Usma Mehmood, Farzana Kausar, Shamim Jaweed, Muhammad Karim, Atiya Hussain, Naila Nadeem, Fyezah Jehan, Sayedur Rahman, Nasreen Islam, Ruksana Azad, Syed Mamum Ibne Moin, Mahmoodur Rahman, Salahuddin Ahmed, Abdul Quiayum, Rasheda Khanam, Abdullah H. Baqui, Sachido Yoshida, Alexander Manu, Rajiv Bahl, Anne CC Lee, Mariam Naqvi, Lauren E. Schaeffer, Rachel Whelan, Blair J. Wylie

## Abstract

**Background:**

We aimed to evaluate and improve the accuracy of the ultrasound scan in estimating gestational age in late pregnancy (ie, after 24 weeks' gestation) in low-income and middle-income countries (LMICs), where access to ultrasound in the first half of pregnancy is rare and where intrauterine growth restriction is prevalent.

**Methods:**

This prospective, population-based, cohort study was done in three LMICs (Bangladesh, Pakistan, and Tanzania) participating in the WHO Alliance for Maternal and Newborn Health Improvement study. Women carrying a live singleton fetus dated by crown-rump length (CRL) measurements between 8^+0^–14^+6^ weeks of gestation, who were willing to return for two additional ultrasound scans, and who planned on delivering in the study area were enrolled in the study. Participants underwent ultrasonography at 24^+0^–29^+6^ weeks and at 30^+0^–36^+6^ weeks' gestation. Birthweights were measured within 72 h of birth, and the proportions of infants who had a small-for-gestational-age birthweight (ie, a birthweight <10% of the standard birthweight for the infant's gestational age and sex according to the INTERGROWTH-21st project newborn baby reference standards) and appropriate-for-gestational-age birthweights were ascertained. Estimation of gestational age by standard fetal biometry measurements in addition to transcerebellar diameter (TCD) measurements was compared with gold-standard CRL measurements by use of Bland-Altman plots to calculate the mean difference and 95% limits of agreement. Statistical modelling was done to develop new gestational age prediction formulas for third trimester ultrasonography in LMICs.

**Findings:**

Between Feb 7, 2015, and Jan 9, 2017, 1947 women were enrolled in the study. 1387 pregnant women had an ultrasound scan at 24^+0^–29^+6^ weeks of gestation and 1403 had an ultrasound scan between 30^+0^–36^+6^ weeks of gestation. Of the 1379 unique infants whose birthweights were available, 981 (71·1%) infants were born with an appropriate-for-gestational-age birthweight and 398 (28·9%) infants were born with a small-for-gestational-age birthweight. The accuracy of late pregnancy ultrasound biometry using existing formulas to estimate gestational age in LMICs was similar to that in high-income settings. With standard dating formulas, late pregnancy ultrasound at 24^+0^–29^+6^ weeks' gestation was accurate to within approximately plus or minus 2 weeks of the gold-standard CRL measurement of gestational age, and late pregnancy ultrasound was accurate to within ±3 weeks of the CRL measurement at 30^+0^–36^+6^ weeks' gestation. In infants who were ultimately born small for gestational age, individual parameters systematically underestimated gestational age, apart from TCD, which showed minimal bias. By use of a novel parsimonious model formula that combined TCD with femur length, gestational age at the 24^+0^ −29^+6^-week ultrasound scan was estimated to within ±10·5 days of the CRL measurement and estimated to within ±15·1 days of the CRL measurement at the 30^+0^–36^+6^-week ultrasound scan. Similar results were observed in infants who were small-for-gestational-age.

**Interpretation:**

Incorporation of TCD and the use of new formulas in late pregnancy ultrasound scans could improve the accuracy of gestational age estimation in both appropriate-for-gestational-age and small-for-gestational-age infants in LMICs. Given the high rates of small-for-gestational-age infants in LMICs, these results might be especially relevant. Validation of this new formula in other LMIC populations is needed to establish whether the accuracy of the late pregnancy ultrasound can be narrowed to within approximately 2 weeks.

**Funding:**

Bill & Melinda Gates Foundation.

**Research in context**

**Evidence before this study**

The performance of late pregnancy ultrasound for assessing gestational age has been understudied in low-income and middle-income countries (LMICs), where access to ultrasound for gestational age dating early in pregnancy is rare and where a high proportion of infants are born small-for-gestational age. In high-income settings, the transcerebellar diameter (TCD) has shown promise as a biometric parameter that is less affected by intrauterine growth restriction than standard ultrasound biometric measurements, but this parameter is not routinely used, as most women in high-income settings present earlier (<20 weeks of gestation) for ultrasound confirmation of gestational age.

**Added value of this study**

In this multicountry, population-based, cohort study, we compared the performance of standard ultrasound biometric parameters and TCD with the gold-standard method of first trimester crown-rump length (CRL) measurements for estimating gestational age in late pregnancy (ie, after 24 weeks' gestation). The accuracy of ultrasonography in estimating gestational age in late pregnancy in LMICs was similar to that reported in high-income settings, but it was further improved when TCD was included in the combination formulas for gestational age assessment. We derived a parsimonious model, requiring measurement of only the TCD and the femur length, that was accurate to within approximately plus or minus 2 weeks of the gold-standard CRL measurement of gestational age, even among infants who were ultimately born small-for-gestational-age. Our study was population-based, enrolling an unselected group of pregnant women rather than a selected group of only the healthiest pregnant women; therefore, we believe our novel gestational age formula is more generalisable to real-world conditions that health-care practitioners in LMICs face.

**Implications of all the available evidence**

In LMICs, gestational age in late pregnancy can be reliably assessed to within approximately ±2 weeks of the CRL measurement by use of only two ultrasound parameters. These results were consistent when our gestational age formulas were applied to pregnancies in which the infants were ultimately born small for gestational age. Our formulas could be most useful when measuring gestational age in pregnant women who present late to receive antenatal care in LMICs, where the burden of intrauterine growth restriction is high. The biometric ultrasound measurements used in our study appear to be attainable by health-care providers with limited sonography training. Accurate gestational age dating can enable providers to identify pregnancies requiring antenatal interventions, such as corticosteroids or transfer to a facility that is able to handle premature infants. Accurate dating will also allow researchers to better estimate the global burden of preterm birth and intrauterine growth restriction.

## Introduction

Pregnancy dating is an essential component of antenatal care. Accurate estimation of gestational age is needed to optimise provision of obstetric interventions and delivery location for preterm births, and it is also a prerequisite to identify and manage fetal growth abnormalities. Additionally, accurate estimation of gestational age is required to provide reasonable global estimates of preterm birth and intrauterine growth restriction.

In low-income and middle-income countries (LMICs), where the burden of preterm birth and intrauterine growth restriction is highest,^1^, ^2^ assessing gestational age accurately can be challenging because of many factors. For instance, last menstrual period is often unknown, and menstrual cycles can vary in length.^3^, ^4^ Symphysis fundal height can be a misleading measure of gestational age because of variation in maternal adiposity, intrauterine growth restriction, uterine fibroids, or malpresentation. The first trimester ultrasound is considered to be the gold-standard method for estimating gestational age in high-income settings,^5^ but ultrasound early in gestation might not be routinely available in LMICs. In addition, pregnant women in LMICs might not present for antenatal care in early pregnancy. As gestation advances, ultrasound biometry becomes less accurate for estimating gestational age, given the emergence of natural variation in fetal size and the possibility of pathological growth restriction. Indeed, in LMICs, where 19·3% of infants are born small-for-gestational-age,^6^ the assumption that fetal size predicts gestational age might not be valid.

Studies^7^, ^8^ suggest that measurements of the cerebellum could allow for a more accurate estimation of gestational age late in pregnancy (ie, from 24 weeks' gestation onwards) compared with standard biometry measurements. Cerebellar size appears to be less affected by intrauterine growth restriction than other measures of growth.^9^ There is also less natural variation in cerebellar size between fetuses. Transcerebellar diameter (TCD) nomograms have been published for several populations,^7^ but they are not in mainstream use in high-income settings, as most women have early ultrasound scans (at <20 weeks' gestation) when standard biometric measurements (biparietal diameter, head circumference, abdominal circumference, and femur length) are reported to provide an accurate estimation of gestational age (to within plus or minus 1–2 weeks of the gold-standard crown-rump length (CRL) measurement of gestational age).^10^ Standard biometry does not perform as well in the third trimester as it does at less than 20 weeks' gestation, with a reported accuracy of only ±3 or more weeks of the CRL measurement.^11^ One study in the USA^9^ found a strong correlation between cerebellar size and gestational age in infants born with an appropriate-for-gestational-age birthweight and in those born small-for-gestational-age. Even though a small number of studies^12^, ^13^ have measured the cerebellum in specific LMICs, the performance of cerebellar biometry for assessing gestational age has been understudied in settings where its use might be of greater benefit.

The objective of the WHO Alliance for Maternal and Newborn Health Improvement (AMANHI) late pregnancy dating study was to evaluate the accuracy of late pregnancy (ie, after 24 weeks' gestation) ultrasound biometry in estimating gestational age in LMIC settings, with a particular focus on the effect of intrauterine growth restriction on the accuracy and whether cerebellar measurements could improve performance.

## Methods

### Study design and participants

The WHO AMANHI study cohort was established in 2010 to harmonise several large ongoing maternal and neonatal health research studies in sub-Saharan Africa and south Asia.^14^ The study involved five distinct sites (Sylhet, Bangladesh; Karachi, Pakistan; Pemba, Tanzania; Brong Ahafo, Gahna; Southern Province, Zambia). Three of the five AMANHI sites (Sylhet, Karachi, and Pemba) participated in this prospective, multicountry, population-based cohort substudy, known as the WHO AMANHI late pregnancy dating study. The remaining two sites (Brong Ahafo and the Southern Province) were ineligible, having completed AMANHI enrolment. AMANHI study populations were predominantly rural with low levels of literacy. As AMANHI was an unselected, population-based cohort, it was inclusive of both appropriately grown and growth-restricted infants.

Between Feb 7, 2015, and Jan 9, 2017, pregnant women were identified by trained field workers through home visits. Urine tests for human chorionic gonadotropin were used to confirm pregnancies. Women were eligible for the study if they were pregnant with a live singleton fetus, the pregnancy had been dated by CRL measurements between 8^+0^ and 14^+0^ weeks' gestation (CRL of <95 mm),^15^ they were willing to return for two additional ultrasounds, and they planned on delivering in the study area. Women pregnant with multiple fetuses, or a non-viable or anomalous fetus were ineligible. Fieldworkers obtained written informed consent from participants in their local languages and arranged study ultrasounds to coincide with standard AMANHI visits.

Ethics approval was granted by institutional review boards and ethics review committees in the participating countries, the host institutions of principal investigators, WHO, and Partners HealthCare (the Boston-based coordinating team).

### Procedures

We considered the first trimester ultrasound scan to be the gold-standard method for estimating gestational age. AMANHI study sonographers followed standardised procedures to obtain high-quality transabdominal CRL measurements. Three CRL measurements were obtained for each participant, and the median CRL value and the INTERGROWTH-21st equation^15^ were used to calculate gestational age. First trimester CRL measurements established the estimated date of delivery and were considered as the gold standard gestational age at all subsequent study visits.

Study ultrasound procedures were standardised during a 4-day training course in Bangladesh for staff from participating AMANHI sites, and consisted of didactic and practical teaching methods.^16^ Competency was assessed with written and practical post-training assessments. Staff from Pakistan were unable to attend because of visa issues and they were therefore trained via Skype conferencing. No additional sonographers were recruited during the study period.

Two ultrasound scans subsequent to the first trimester ultrasound were done for each participant: the first, between 24^+0^ and 28^+6^ weeks' gestation, and the second, between 32^+0^ and 36^+6^ weeks' gestation, both coinciding with routine AMANHI visits. After study initiation, the gestational age windows were extended to 24^+0^–29^+6^ weeks and 30^+0^–36^+0^ weeks to cover a wider range of the third trimester; however, only the Pemba site received ethics approval for this extension before study completion. At every ultrasound visit, sonographers followed standardised procedures to obtain high-quality measurements of the biparietal diameter, head circumference, abdominal circumference, femur length, and TCD (appendix p 4).

Sonographers were masked to the gold-standard gestational age established by CRL measurements. Ultrasound biometry measurements were obtained without the use of an automated biometry software package, so that only absolute measurements were taken. Two measurements for each biometric parameter were obtained and averaged. Plausible measurement ranges were defined for each parameter before the analysis (CRL 2·0–95·0 mm; biparietal diameter 3·0–11·0 cm; head circumference 10·0–40·0 cm; femur length 2·0–9·0 cm; abdominal circumference 12·0–42·0 cm; and TCD 1·5–6·0 cm). For measures outside of these ranges, data were checked for data entry errors and were assigned as missing if unresolved. Gestational age was calculated from each individual biometric parameter by use of standard tables (Hadlock and colleagues^17^ for biparietal diameter, femur length, and abdominal circumference; Chavez and colleagues^18^ for TCD; and INTERGROWTH-21st project^19^ for head circumference). Gestational age was also calculated by use of previously published formulas that combine more than one parameter (Hadlock and colleagues^17^ and INTERGROWTH-21st project^19^). Formulas and the reasonable ranges for individual parameters are detailed in the appendix (p 2).

An image quality checklist provided objective guidance on appropriate image magnification, ideal image plane, and correct calliper placement. All study images were reviewed by BJW, who was masked to the gold-standard gestational age values, for ongoing quality control with quarterly feedback to the sonographers.

Infants were visited within 72 h of birth. Birthweights were obtained by use of digital scales. Small-for-gestational-age was defined as less than 10% of the standard birthweight for the infant's gestational age and sex according to the INTERGROWTH-21st project newborn baby reference standards.^20^, ^21^

### Statistical analysis

Study sample size was calculated on the basis of our hypothesis that the gestational age agreement between TCD and CRL would be higher than the agreement between standard biometry and CRL, both among infants with appropriate-for-gestational-age birthweights and among those with small-for-gestational-age birthweights. Previously published literature^11^ and professional society guidelines^5^ have shown that late pregnancy biometry combination formulas have a gestational age accuracy of within ±21–30 days of the gold-standard first trimester CRL measurements. By use of overlapping Bland-Altman plots to compare the degree of agreement between TCD versus CRL with standard biometry versus CRL, a sample size of 50 would be adequate to show a mean difference of 0 and 95% limits of agreement (LOA) of within 10 days (TCD *vs* CRL) versus within 21 days (standard biometry *vs* CRL) of the CRL-estimated gestational age. We aimed to enrol a minimum of 50 infants with small-for-gestational-age birthweights per site.^22^, ^23^ Assuming that 20% of infants would be small-for-gestational-age and that 25% of pregnant women would be lost to follow-up at delivery, we required 333 infants per site; therefore, we had a conservative target of enrolling 400 participants per study site.

The Stata software programme (StataCorp, Stata Statistical Software: Release 14.1; College Station, TX, USA) was used for all statistical analyses. Bland-Altman plots were used to assess the agreement between gestational age measured by gold-standard CRL and gestational age measured by late pregnancy ultrasound biometric parameters.^24^ Mean differences (bias) with 95% CIs and 95% LOA values were calculated for each comparison. For ease of comparison, an approximation of the degree of agreement was calculated as the midpoint between the unsigned LOA values. As an example, if the 95% LOA was −20 to +22 days, the approximation of agreement would be within ±21 days of the gestational age, as determined by the gold-standard first trimester CRL measurement. For individual biometric parameters or combinations of parameters with bias (ie, if the 95% CI of the mean difference does not include 0), the gestational age window agreement limits would be skewed in the direction of the bias.

For infants with available birthweights, we stratified the results by infant size to compare the accuracy of late pregnancy biometry measures among infants with appropriate-for-gestational-age birthweights and small-for-gestational-age birthweights.

Statistical modelling was used to develop novel equations to predict gestational age with late pregnancy biometric parameters as covariates. Generalised linear regression was done by use of the Stata glm command, accounting for clustering of multiple visits for a participant. Gold-standard gestational age determination by CRL measurement was the dependent variable. Covariates tested in the models included all late pregnancy biometric parameters measured (biparietal diameter, head circumference, femur length, abdominal circumference, and TCD). Natural logarithms were used to transform continuous measures given non-normal distributions. Models with individual and combinations of biometric parameters were tested. Given the performance of TCD as an individual parameter, models including TCD as the main predictor were examined, adding additional biometric parameters, assessing for significance (ie, if the p value was <0·05) and improvement in the adjusted R^2^, and determining the best-fit model according to the Akaike Information Criterion.

Potential models were cross-validated to compare the predictive accuracy of the different models and select biometric parameters to be included in the final model. Two methods were used to cross-validate the models. In the first method, the study dataset was divided into five random subsamples, one subsample was withheld, and statistical models were generated with the remaining 80% of the data. The model was then used to predict gestational age in the withheld 20% subsample, and a standardised error term was calculated on the basis of the predicted gestational age, calculated as: (observed gestational age – predicted gestational age)^2^/(predicted gestational age). This process was repeated with each successive withheld subsample, and a summary measure of the prediction error was generated for the entire dataset. The model with the lowest prediction error was selected as the final model. In the second cross-validation method, the data from one study site were withheld, and statistical models were generated with data from the remaining two sites. The model was then fit and used to predict gestational age in the first site that had the data withheld. This process was repeated twice and the standardised error was calculated.

### Role of the funding source

The funder of the study had no role in the study design, data collection, data analysis, data interpretation, or writing of the report.

## Results

**Figure 1:**
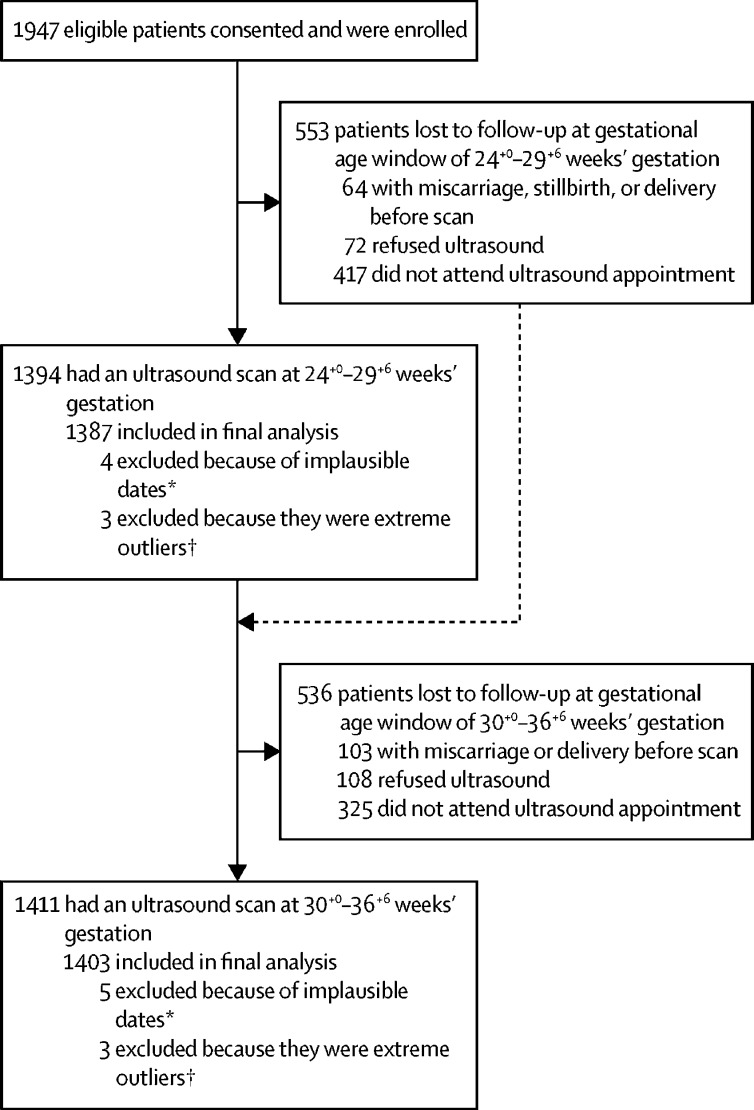
Participant flowchart by ultrasound visit *Exclusion due to implausible dates that could not be reconciled (eg, date of birth before date of ultrasound, or year of ultrasound or birth recorded incorrectly). † Extreme outliers refers to cases in which a difference of greater than 60 days between the gold-standard measurement of gestational age and the gestational age predicted by late pregnancy biometry measurements was observed.

Between Feb 7, 2015, and Jan 9, 2017, 1947 pregnant women in three of the five AMANHI study sites (Bangladesh, Pakistan, and Tanzania) with documented CRL assessments between 8^+0^ and 14^+0^ weeks' gestation were enrolled in this late pregnancy ultrasound study. Of these women, 1619 completed late pregnancy scans. Data from nine infants were excluded from the final analysis because of implausible dates that could not be reconciled (eg, date of birth before date of ultrasound, or year of ultrasound or birth recorded incorrectly). We determined a priori that a difference of greater than 60 days between the gold-standard CRL measurement of gestational age and the gestational age predicted by late pregnancy biometry measurements was considered to be implausibly associated with the performance of the ultrasound. Three such extreme outliers were excluded at 24^+0^–29^+6^ weeks' gestation, and an additional three were excluded at 30^+0^–36^+6^ weeks' gestation. The final analysis included 1611 unique mothers, of whom 1387 underwent an ultrasound scan between 24^+0^ and 29^+6^ weeks' gestation (546 in Bangladesh, 456 in Pakistan, and 385 in Tanzania), and 1403 underwent an ultrasound scan between 30^+0^ and 36^+6^ weeks' gestation (531 in Bangladesh, 449 in Pakistan, and 423 in Tanzania; figure 1).

More than 97% of all attempts at biometric measurements were successful. Of the 1387 ultrasound scans done at 24^+0^–29^+6^ weeks' gestation, values for the biparietal diameter were missing in two (0·1%) scans, for head circumference in three (0·2%) scans, for femur length in two (0·1%) scans, for abdominal circumference in three (0·2%) scans, and for TCD in three (0·2%) scans. Of the 1403 ultrasound scans done at 30^+0^–36^+6^ weeks' gestation, missing values for the different parameters were similar to the ultrasound scan at 24^+0^–29^+6^ weeks' gestation, with two (0·1%) values missing for biparietal diameter, two (0·1%) for head circumference, none for femur length, and eight (0·6%) for abdominal circumference. Even at 30^+0^–36^+6^ weeks' gestation, TCD measurements were successfully obtained from 1365 (97%) of 1403 ultrasound scans. Summary measures from the individual biometric parameters (mean and SD) for the cohort and by site are included in the appendix (p 1). Using the reference nomogram for CRL by Hadlock and colleagues^17^ as an alternative to the INTERGROWTH-21st project^19^ nomogram, no significant differences in the distribution of gestational ages estimated from either third trimester ultrasound scan were observed (data not shown).

**Figure 2:**
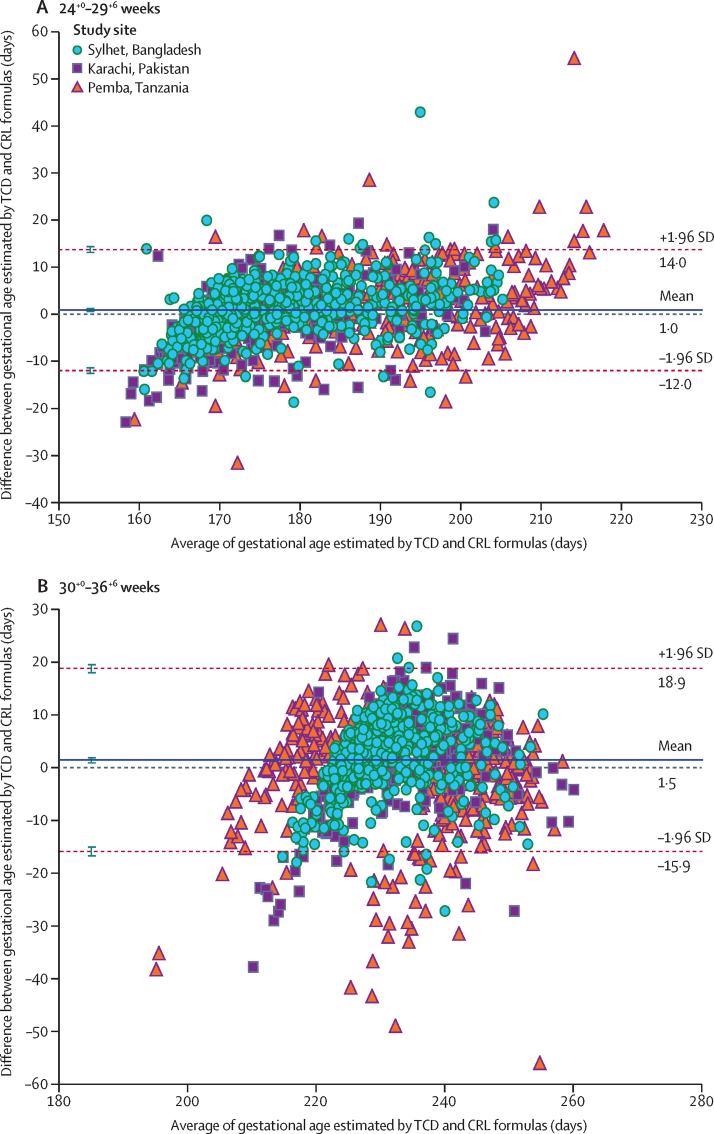
Bland-Altman plots comparing agreement of gestational age (days) determied by TCD measurements with first trimester CRL measurements at 24^+0^–29^+6^ weeks' gestation (A) and 30^+0^–36^+6^ weeks' gestation (B), by study site The identity line at y=0 represents values for which the TCD and CRL measurements would yield the same unbiased estimate of gestational age. The mean difference (ie, bias) is the average difference between the two measurements and is plotted as a solid line. The 95% limits of agreement between the two methods are represented by the dashed lines. TCD=transcerebral diameter. CRL=crown-rump length.

From the ultrasound scans done at 24^+0^–29^+6^ weeks' gestation, the performance of individual biometric parameters showed fairly similar agreement with gold-standard CRL measurements (approximations ranging from ±12·8 to ±16·2 days of the standard CRL measurements; table 1). There was little bias, as evidenced by a mean difference close to zero for all parameters. A representative Bland-Altman plot for TCD versus CRL is presented in figure 2.

**Table 1:** Degree of agreement between late pregnancy ultrasound and gold-standard first trimester crown-rump length measurement of gestational age

	**Ultrasound scan at 24^+0^–29^+^ weeks' gestation (n=1387)**			**Ultrasound scan at 30^+0^–36^+^ weeks' gestation (n=1403)**		
	Mean difference in days (95% CI)	95% limits of agreement in days	Approximation in days*	Mean difference in days (95% CI)	95% limits of agreement in days	Approximation in days*
**Individual parameters**						
TCD^18^	1·0 (0·6 to 1·3)	−12·0 to 14·0	±13·0	1·5 (1·0 to 2·0)	−15·9 to 18·9	±17·4
BPD^17^	−2·6 (−2·9 to −2·2)	−17·0 to 11·9	±14·5	−8·0 (−8·6 to −7·4)	−30·3 to 14·3	±22·3
HC^19^	1·5 (1·2 to 1·9)	−13·0 to 16·1	±14·6	0·7 (−0·0 to 1·3)	−24·5 to 25·9	±25·2
FL^17^	−2·1 (−2·4 to −1·7)	−14·9 to 10·7	±12·8	−7·7 (−8·2 to −7·2)	−27·5 to 12·1	±19·8
AC^17^	−0·2 (−0·7 to 0·2)	−16·4 to 16·0	±16·2	−4·8 (−5·4 to −4·1)	−29·0 to 19·4	±24·2
**Combination formulas**						
Hadlock^17^	−3·2 (−3·5 to −2·9)	−14·3 to 7·9	±11·1	−7·7 (−8·1 to −7·2)	−25·6 to 10·3	±18·0
INTERGROWTH-21st project^19^	1·5 (1·2 to 1·8)	−10·4 to 13·4	±11·9	0·8 (0·3 to 1·3)	−19·0 to 20·6	±19·8
AMANHI parsimonious formula	1·2 (0·9 to 1·4)	−9·3 to 11·6	±10·5	−1·4 (−1·8 to −1·0)	−16·5 to 13·7	±15·1

The Hadlock formula^17^ includes BPD, AC, and FL measurements; INTERGROWTH-21st project formula^19^ includes HC and FL measurements; and the AMANHI parsimonious method includes TCD and FL measurements. TCD=transcerebellar diameter. BPD=biparietal diameter. HC=head circumference. FL=femur length. AC=abdominal circumference. AMANHI=Alliance for Maternal and Newborn Health Improvement.

* Approximation refers to the midpoint between the unsigned limits of agreement, and values represent the number of days plus or minus the gold-standard first trimester crown-rump length measurement of gestational age; for biometric parameters or combinations with bias (ie, if the 95% CI of the mean difference does not include 0), the true value of gestational age compared with the estimated value from third trimester biometry will be skewed in the direction of the bias (mean difference).

From the ultrasound scans done at 30^+0^–36^+6^ weeks' gestation, TCD had narrower 95% LOA than the other individual biometric parameters (table 1). The agreement between TCD and gold-standard CRL measurements was approximated to be within ±17·4 days of the CRL measurement with little bias (mean difference 1·5 days, 95% CI 1·0–2·0). Compared with TCD, the remaining individual parameters showed less agreement with CRL measurements (approximations ranged between ±19·8–25·2 days). A strong negative bias for biparietal diameter (–8·0 days, 95% CI −8·6 to −7·4), femur length (–7·7 days, −8·2 to −7·2), and abdominal circumference (–4·8 days, −5·4 to −4·1) was observed, suggesting a systematic underestimation of gestational age by approximately 1 week.

From the ultrasound scan at 24^+0^–29^+6^ weeks' gestation, the Hadlock^17^ and INTERGROWTH-21st project^19^ combination formulas outperformed individual parameters by a few days (approximations ranged between ±11·1–11·9 days) and maintained little bias (table 1). From the ultrasound scan at 30^+0^–36^+6^ weeks' gestation, TCD alone (approximation ±17·4 days) showed a similar degree of agreement to CRL measurements as the Hadlock^17^ formula (approximation ±18·0 days), and slightly narrower agreement with CRL measurements than the INTERGROWTH-21st project^19^ formula (approximation 19·8 days). Notably, the Hadlock^17^ formula had a strong negative bias (mean difference −7·7 days, 95% CI −8·1 to −7·2). Results did not vary substantially by site (data not shown) and have been presented together.

Stratification of results by infant size was done in women who were less than 42 weeks pregnant when they gave birth with birthweights available, and included 1186 (86%) of 1387 women who had an ultrasound scan at 24^+0^–29^+6^ weeks' gestation, 1236 (88%) of 1403 women who had an ultrasound scan at 30^+0^–36^+6^ weeks' gestation, and 1379 (86%) of all 1611 unique infants for whom the birthweights were known. 398 (29%) of the 1379 unique infants were classified as small-for-gestational-age. The prevalence of infants who were small-for-gestational-age varied by site and was higher in Asian sites (234 [44%] of 526 infants in Bangladesh and 122 [28%] of 430 infants in Pakistan) than in the African sites (42 [10%] of 423 infants in Tanzania).

TCD outperformed the other biometric parameters in predicting gestational age and showed less negative bias (ie, less underestimation), with 95% LOA values that were similar at the 24^+0^–29^+6^-week and 30^+0^–36^+6^-week scans in infants who had appropriate-for-gestational age birthweights and small-for-gestational-age birthweights (table 2). Hadlock^17^ and INTERGROWTH-21st project^19^ combination formulas had slightly narrower 95% LOA values than individual parameters at the 24^+0^–29^+6^-week scan in infants who were small-for-gestational-age (table 2), although the Hadlock^17^ formula had substantial negative bias (–5·7 days, 95% CI −6·2 to −5·2). At the 30^+0^–36^+6^-week scan, TCD had a similar 95% LOA to the combination formulas for infants who were small-for-gestational-age (approximation 15·8 days) but with less bias (mean difference 0·3 days) than both the Hadlock^17^ (–13·2 days) and INTERGROWTH-21st project^19^ formulas (–4·2 days; table 2).

**Table 2:** Performance of late pregnancy ultrasound in estimating the gestational age of infants with small-for-gestational-age or appropriate-for-gestational-age birthweights

	**Birthweight appropriate for gestational age**			**Birthweight small for gestational age**		
	Mean difference in days (95% CI)	95% limits of agreement in days	Approximation in days*	Mean difference in days (95% CI)	95% limits of agreement in days	Approximation in days*
**24^+0^–29^+6^ weeks' gestation**						
TCD^18^	1·4 (1·0 to 1·9)	−12·1 to 15·0	±13·6	0·3 (−0·4 to 0·9)	−11·5 to 12·1	±11·8
BPD^17^	−1·6 (−2·1 to −1·1)	−16·0 to 12·8	±14·4	−4·7 (−5·4 to −4·0)	−17·9 to 8·5	±13·2
HC^19^	3·1 (2·6 to 3·6)	−11·4 to 17·5	±14·5	−1·9 (−2·5 to −1·2)	−13·5 to 9·8	±11·7
FL^17^	−1·1 (−1·5 to −0·6)	−13·9 to 11·8	±12·9	−3·8 (−4·4 to −3·1)	−15·1 to 7·6	±11·4
AC^17^	1·6 (1·1 to 2·2)	−14·3 to 17·6	±16·0	−4·1 (−4·8 to −3·3)	−17·7 to 9·6	±13·7
Hadlock^17^	−1·9 (−2·3 to −1·5)	−12·7 to 9·0	±10·9	−5·7 (−6·2 to −5·2)	−15·1 to 3·7	±9·4
INTERGROWTH-21st project^19^	2·9 (2·5 to 3·3)	−8·9 to 14·6	±11·8	−1·1 (−1·6 to −0·6)	−10·7 to 8·4	±9·6
AMANHI parsimonious formula	1·9 (1·5 to 2·3)	−8·8 to 12·5	±10·7	0·1 (−0·4 to 0·6)	−9·1 to 9·2	±9·2
**30^+0^–36^+6^ weeks' gestation**						
TCD^18^	1·9 (1·3 to 2·6)	−16·2 to 20·1	±18·2	0·3 (−0·5 to 1·2)	−15·4 to 16·1	±15·8
BPD^17^	−5·7 (−6·4 to −5·0)	−27·1 to 15·7	±21·4	−13·2 (−14·2 to −12·1)	−33·7 to 7·4	±20·6
HC^19^	3·6 (2·7 to 4·4)	−21·1 to 28·2	±24·7	−5·8 (−6·9 to −4·7)	−26·7 to 15·2	±21·0
FL^17^	−5·9 (−6·5 to −5·2)	−25·0 to 13·3	±19·2	−11·3 (−12·2 to −10·4)	−28·2 to 5·5	±16·9
AC^17^	−1·1 (−1·9 to −0·4)	−22·9 to 20·6	±21·8	−12·9 (−14·0 to −11·8)	−33·3 to 7·5	±20·4
Hadlock^17^	−5·1 (−5·6 to −4·5)	−21·1 to 11·0	±16·1	−13·2 (−14·0 to −12·4)	−28·5 to 2·1	±15·3
INTERGROWTH-21st project^19^	3·2 (2·6 to 3·9)	−15·5 to 22·0	±18·8	−4·2 (−5·1 to −3·4)	−20·8 to 12·3	±16·6
AMANHI parsimonious formula	−0·3 (−0·9 to 0·2)	−15·6 to 14·9	±15·3	−3·7 (−4·4 to −3·0)	−16·7 to 9·3	±13·0

The Hadlock formula^17^ includes BPD, AC, and FL measurements; INTERGROWTH-21st project formula^19^ includes HC and FL measurements; and the AMANHI parsimonious method includes TCD and FL measurements. TCD=transcerebellar diameter. BPD=biparietal diameter. HC=head circumference. FL=femur length. AC=abdominal circumference. AMANHI=Alliance for Maternal and Newborn Health Improvement.

* Approximation refers to the midpoint between the unsigned limits of agreement and values represent the number of days plus or minus the gold-standard first trimester crown-rump length measurement of gestational age; for biometric parameters or combinations with bias (ie, if the 95% CI of the mean difference does not include 0), the true value of gestational age compared with the estimated value from third trimester biometry will be skewed in the direction of the bias (mean difference).

The four statistical models generated from the AMANHI late pregnancy dating study are shown in table 3. Model 1 was the most accurate in predicting gestational age and included four biometric parameters. Model 2 included only TCD and femur length and showed the least prediction error among the three more parsimonious models (combining only two parameters) after both cross-validation approaches were applied (appendix p 3).

**Table 3:** Performance of newly derived Alliance for Maternal and Newborn Health Improvement combination formulas for determining gestational age after 24 weeks' gestation

	**24^+0^–29^+6^ weeks' gestation**			**30^+0^–36^+6^ weeks' gestation**		
	Mean difference in days (95% CI)	95% limits of agreement in days	Approximation in days*	Mean difference in days (95% CI)	95% limits of agreement in days	Approximation in days*
Model 1: lnTCD, lnBPD, lnFL, and lnAC†	1·1 (0·9 to 1·4)	−8·9 to 11·2	±10·1	−1·4 (−1·7 to −1·0)	−16·0 to 13·3	±14·7
Model 2: lnTCD and lnFL‡	1·2 (0·9 to 1·4)	−9·3 to 11·6	±10·5	−1·4 (−1·8 to −1·0)	−16·5 to 13·7	±15·1
Model 3: lnTCD and lnBPD	1·4 (1·1 to 1·6)	−9·5 to 12·2	±10·9	−1·6 (−2·0 to −1·2)	−17·8 to 14·6	±16·2
Model 4: lnTCD and lnAC	1·3 (1·0 to 1·6)	−9·5 to 12·1	±10·8	−1·6 (−2·0 to −1·1)	−17·8 to 14·6	±16·2

TCD=transcerebellar diameter. BPD=biparietal diameter. FL=femur length. AC=abdominal circumference. HC=head circumference.

* Approximation refers to the midpoint between the unsigned limits of agreement and values represent the number of days plus or minus the gold-standard first trimester crown-rump length measurement of gestational age; for biometric parameters or combinations with bias (ie, if the 95% CI of the mean difference does not include 0), the true value of gestational age compared with the estimated value from third trimester biometry will be skewed in the direction of the bias (mean difference).

† Model 1 is the complete Alliance for Maternal and Newborn Health Improvement model.

‡ Models 2–4 evaluate combinations of fewer biometric parameters, with Model 2 chosen as the Alliance for Maternal and Newborn Health Improvement parsimonious model.

The final model was fit by including TCD and femur length in a regression with the entire dataset (model 2, table 3; formula in appendix p 3). The prediction error (appendix p 3) is on the final model involving the entire cohort data. Formulas for all candidate models and their out-of-model prediction errors are shown in the appendix (p 3).

From the ultrasound scan at 24^+0^–29^+6^ weeks' gestation, our novel AMANHI combination formula, combining TCD and femur length measurements, showed little improvement in the accuracy of estimating gestational age when infants were stratified by birthweight (appropriate-for-gestational-age or small-for-gestational-age) compared with previous combination formulas,^17^, ^19^ improving estimations by only 1 day (table 2). However, from the ultrasound scan at 30^+0^–36^+6^ weeks' gestation, the AMANHI combination formula could more accurately predict gestational age for appropriate-for-gestational-age infants and small-for-gestational-age infants compared with the INTERGROWTH-21st project^19^ combination formula, narrowing the 95% LOA to approximately ±2 weeks and with minimal bias (table 2).

## Discussion

We have identified several key findings regarding the performance of ultrasound biometry in late pregnancy in a large cohort of pregnant women in three LMIC settings. First, it was reassuring to verify that the accuracy of late pregnancy ultrasound for estimating gestational age in our study was similar to the reported results from higher-income settings, where sonographer training is lengthier and includes more practical training compared with sonographer training in LMIC settings. Biometric parameters at 24^+0^–29^+6^ weeks' gestation and 30^+0^–36^+6^ weeks' gestation, alone and in combination, were accurate to within approximately ±3·0–3·5 weeks of the gold-standard CRL measurement of gestational age. These results are consistent with previous reports of the performance of late pregnancy biometric parameters.^22^, ^23^, ^24^

In our study, the accuracy of gestational age dating with individual biometric parameters before 30 weeks was reasonably good (approximation ±12·8 days to ±16·2 days of the standard CRL measurements), improving even further (approximation ±11·1–11·9 days) when individual biometric parameters were combined according to previously published formulas. Later scans (>30 weeks) had wider individual 95% LOAs (approximation ±17·4–25·2 days). As hypothesised, the TCD, as a single biometric parameter, had the narrowest 95% LOA (approximation ±17·4 days), and even outperformed the combination formulas slightly (approximation ±18·0–19·8 days). Importantly, TCD also performed quite well among infants who were ultimately born small-for-gestational-age with an accuracy of approximately ±11·8 days of the CRL measurement of gestational age at 24^+0^–29^+6^ weeks and approximately ±15·8 days of the CRL measurement at 30^+0^–36^+6^ weeks, with little bias (mean difference of 0·3 days at both gestational age windows). In summary, nomograms established for measuring cerebellar size in high-resource settings appear to perform well in this LMIC population.

Given the initial results showing successful performance of TCD in estimating gestational age, we combined TCD with standard biometric parameters and used statistical modelling to investigate the performance of different combination formulas in the LMIC study sites, where the prevalence of infants born small-for-gestational-age is high. Our first model, combining TCD with biparietal diameter, femur length, and abdominal circumference, narrowed the 95% LOA across the two third trimester gestational age windows to less than 2 weeks. A second parsimonious model, combining only TCD and femur length, performed nearly as well as the first model, with the added benefit of simplicity in the number of parameters included. Both the femur and the cerebellum are structures that are fairly easy to identify. In addition, the measurements of these parameters are linear, and therefore require less skill in calliper placement than circumference measurements, as evidenced by the observation that these measurements were obtained successfully in 97% of ultrasound scans in our study. When compared with other published combination formulas, our AMANHI parsimonious model formula had the narrowest 95% LOA, the least bias, and performed similarly among infants born appropriate for gestational age and small for gestational age in both gestational age windows. As the AMANHI formulas were derived from our study population, it is not surprising that they performed well, even after attempting to separate model development and validation through cross-validation exercises. Our gestational age formulas should therefore be viewed as hypothesis-generating, and they should be validated in other LMIC populations before they are used extensively.

It is important to emphasise that our study included an unselected and population-based cohort from three LMICs at the time of enrolment. The cohort therefore reflects the range of morbidities associated with pregnancy, maternal nutritional status, and fetal sizes that would be encountered by a health-care practitioner in similar settings. Our cohort contrasts those used in previous studies,^19^, ^20^, ^21^ such as the INTERGROWTH-21st project^19^ cohort, that were designed to measure optimal fetal growth and enrolled only healthy low-risk women who were less likely to carry a growth-restricted fetus. These differences might help to explain why our AMANHI parsimonious model outperformed both the Hadlock^17^ and INTERGROWTH-21st project^19^ formulas among infants who were born small for gestational age. Even at 30^+0^–36^+6^ weeks' gestation, the 95% LOA of our AMANHI parsimonious formula was still within ±15 days of the CRL measurement of gestational age, and it performed similarly among infants born with small-for-gestational-age (approximation ±13·0 days) or appropriate-for-gestational-age (approximation ±15·3 days) birthweights. An estimation of gestational age to within approximately ±2 weeks of the standard CRL measurement is a remarkable achievement for LMIC settings, as many women do not have an ultrasonography in the first half of pregnancy. If intrauterine growth restriction is suspected, measurement of cerebellar size in the third trimester could help to confirm that this is the case.

Our study has several strengths, including its large sample size and the determination of gestational age by use of gold-standard first trimester CRL measurements that were verified by a multilayered quality control process. Nonetheless, there are some limitations of our study. We did not evaluate the feasibility of obtaining cerebellum measurements after 36 weeks' gestation. With advancing gestation, shadowing from the calcified skull might preclude the accurate measurement of the TCD margins, limiting its use as the pregnancy nears full term. The results shown in figure 2 suggest that accurate TCD measurements might be more difficult to obtain as the pregnancy advances, as several individual data points fell below the lower 95% LOA rather than the upper LOA at the 30^+0^–36^+6^-week gestational age window. This underestimation of gestational age by TCD in this gestational age window could reflect difficulties in measuring the full size of the cerebellar hemispheres.

In summary, if validated in other LMIC populations, our novel parsimonious model for estimating gestational age in late pregnancy could be useful for research, public health surveillance, and clinical care. Our study findings might be clinically relevant for health-care providers tasked with determining whether a fetus is small rather than preterm, and thus might limit iatrogenic harm from unnecessary preterm delivery.

Two important messages can be concluded from our work. First, cerebellar measurements using previously established nomograms from high-resource settings are a useful adjunct to late pregnancy biometry measurements in LMICs, where the burden of infants born small-for-gestational-age is high. Secondly, if validated in other LMIC populations, a parsimonious gestational age formula requiring measurement of only two biometric parameters might narrow accuracy of late pregnancy ultrasound to within approximately 2 weeks.

Correspondence to: Blair J Wylie, Division of Maternal-Fetal Medicine, Beth Israel Deaconess Medical Center, Harvard Medical School, Boston, MA 02215, USA bwylie@bidmc.harvard.edu

or

Rajiv Bahl, Department of Maternal, Newborn, Child and Adolescent Health, WHO, Geneva, 1211, Switzerland bahlr@who.int
