# Correction to *Lancet Glob Health* 2020; 8: e545–54

**DOI:** 10.1016/S2214-109X(20)30473-3

**Published:** 2020-11-05

T*he WHO Alliance for Maternal and Newborn Health Improvement Late Pregnancy Dating Study Group. Performance of late pregnancy biometry for gestational age dating in low-income and middle-income countries: a prospective, multicountry, population-based cohort study from the WHO Alliance for Maternal and Newborn Health Improvement (AMANHI) Study Group.* Lancet Glob Health *2020; **8:** e545–54*—In the appendix of this article, the reasonable range of the crown-rump length parameter in eTable 2 should have been 2·0–95·0 mm. In addition, the unit of measurement for biparietal diameter, femur length, head circumference, and transcerebellar diameter covariates in eTable 3 was not initially included and should have been millimetres. The appendix of this article has been corrected as of Nov 5, 2020.

