# Supplementary material for: Performance of late pregnancy biometry for gestational age dating in low-income and middle-income countries: a prospective, multicountry, population-based cohort study from the WHO Alliance for Maternal and Newborn Health Improvement (AMANHI) Study Group
Source: Lancet Glob Health. 2020 Mar 18;8(4):e545–54. doi: 10.1016/S2214-109X(20)30034-6 (PMC7091029; doi:10.1016/S2214-109X(20)30034-6)
Supplement: Supplementary appendix [file mmc1.pdf]

THE LANCET
Global Health

Supplementary appendix

This appendix formed part of the original submission and has been peer reviewed. We post it as supplied by the authors.

Supplement to: The WHO Alliance for Maternal and Newborn Health Improvement Late Pregnancy Dating Study Group. Performance of late pregnancy biometry for gestational age dating in low-income and middle-income countries: a prospective, multicountry, population-based cohort study from the WHO Alliance for Maternal and Newborn Health Improvement (AMANHI) Study Group. *Lancet Glob Health* 2020; **8:** e545–54.

SUPPLEMENTARY MATERIAL

Performance of late pregnancy biometry for gestational age dating in low-income and middle-income countries: a prospective, multicountry, population-based cohort study from the WHO Alliance for Maternal Newborn Health Improvement (AMANHI) study group

eTable 1: Summary of biometric parameter measurements, by gestational age window and by site.

	Sylhet, Bangladesh	Karachi, Pakistan	Pemba, Tanzania	Total Cohort
	Mean (SD)	Mean (SD)	Mean (SD)	Mean (SD)
Birthweight (g)	2704.7 (440.5)	2821.6 (460.7)	3344.0 (500.9)	2943.3 (542.2)
**24-29^{+6} Weeks**				
CRL (cm)	4.8 (1.8)	4.4 (1.9)	4.5 (1.9)	4.6 (1.9)
TCD (cm)	2.9 (0.3)	2.7 (0.2)	3.1 (0.3)	2.9 (0.3)
BPD (cm)	6.0 (0.5)	6.0 (0.4)	6.5 (0.6)	6.2 (0.5)
HC (cm)	22.8 (1.6)	23.1 (1.5)	25.0 (2.0)	23.5 (1.9)
FL (cm)	4.5 (0.4)	4.4 (0.3)	4.9 (0.4)	4.6 (0.4)
AC (cm)	20.1 (1.5)	20.5 (1.7)	22.4 (2.1)	20.9 (2.0)
**30-36^{+0} Weeks**				
CRL (cm)	4.8 (1.8)	4.4 (1.9)	4.5 (1.8)	4.6 (1.9)
TCD (cm)	4.2 (0.3)	4.3 (0.3)	4.3 (0.5)	4.3 (0.4)
BPD (cm)	7.7 (0.4)	8.1 (0.4)	8.1 (0.5)	7.9 (0.5)
HC (cm)	29.2 (1.2)	30.4 (1.4)	30.9 (1.9)	30.1 (1.7)
FL (cm)	6.0 (0.3)	6.2 (0.3)	6.3 (0.5)	6.2 (0.4)
AC (cm)	27.1 (1.7)	28.6 (2.2)	29.6 (2.7)	28.3 (2.4)

eTable 2: Ultrasound reference curves for biometric parameters alone and in combination

Parameter Abbreviation	Definition	Reference	Reasonable Range	Equation/Formula for GA in Days (Parameters measured in cm or mm, as noted)
CRL	Crown-Rump Length (CRL)	INTERGROWTH-21st Project 2014[1]	2-9.5cm	GA=40.9041+(3.21585*(√CRL))+(0.348956*CRL); SD of GA=2.39102+(0.0193474*CRL); GA in days and CRL in mm
TCD	Transcerebellar Diameter (TCD)	Chavez 2004[2]	1.5-6cm	$GA=7*(8.119+(4.244*(TCD))+(1.113*(TCD)^2) – (0.169*(TCD)^3))$; SD of $GA=7*(1.713-1.304(TCD)+0.576(TCD)^2 -0.067(TCD^3))$; GA in days, TCD in cm
BPD	Biparietal Diameter (BPD, Outer-to-Inner)	Hadlock 1984[3]	3-11 cm	$GA=7*((9.54+1.482*BPD) + (0.1676*BPD^2))$; SD=1.36+0*7; GA in days, BPD in cm
HC	Head Circumference (HC)	INTERGROWTH-21st Project 2016[4]	10-40cm	$GA=exp(0.05970*(ln(HC)^2) +0.000000006409*HC^3 +3.3258)$; $SD=0.6492*(GA*0.01)^3 +2.991$; GA in days, HC in mm
FL	Femur Length (FL)	Hadlock 1984[3]	2-9cm	$GA=7*(10.35+(2.46*FL)+(0.17*FL^2)$; SD=1.28*7; GA in days, FL in cm
AC	Abdominal Circumference (AC)	Hadlock 1984[3]	12-42cm	$GA=7*(8.14+0.753(AC)+0.0036+0(AC)^2)$; SD=1.31*7; GA in days, AC in cm
Hadlock Combo	Hadlock Combination Formula (BPD, FL, AC)	Hadlock 1984[3]	As above	GA=7*(10.61+(0.175*BPD*FL) + (0.297*AC) + (0.71*FL)); SD=1.06*7; GA in days, all in cm
INTERGROWTH-21st Project Combo	INTERGROWTH-21st Project Combination Formula (HC, FL)	INTERGROWTH-21st Project 2016[4]	As above	$GA=exp(0.03243*(ln(HC))^2 +0.001644*FL*ln(HC) +3.813)$; SD=0.04009*GA-1.149; GA in days, FL and HC in mm

eTable 3. Performance of candidate WHO AMANHI late pregnancy gestational age formulas

Model	Co-variates	Formula	Adjusted R^2	AIC	Out of Model Prediction Error[a]	Out of Model Prediction Error[b]
1	TCD, FL, BPD, AC	lnGA= 0.3107083(lnTCD)+0.2450894(lnFL)+ 0.1397663(lnBPD)+0.0626322(lnAC)+ 2.297582	0.9517	-4.120	614.5	685.1
2[c]	TCD, FL	lnGA= 0.3825021(lnTCD)+0.3321277(lnFL)+ 2.63416	0.9481	-4.048	673.3	690.6
3	TCD, BPD	lnGA= 0.4390124(lnTCD)+0.2968778(lnBPD) + 2.490502	0.9413	-3.925	735.9	787.6
4	TCD, AC	lnGA= 0.4569856(lnTCD)+0.2244807(lnAC)+ 2.454795	0.9412	-3.924	730.2	779.2

BPD= biparietal diameter; FL=femur length; HC= head circumference; LOA=Limits of agreement; TCD= transcerebellar diameter.
lnGA predicted in days.

[a] Cross Validation by withholding random 20% of data: Sum of (Observed GA -predicted GA)^2/predicted in validation data set (days)

[b] Cross Validation by withholding data from one site.

[v] Model 2 is the AMANHI parsimonious model.

PROTOCOL FOR MEASUREMENT OF THE TRANSCEREBELLAR DIAMETER

The transcerebellar diameter (TCD_ is the maximal diameter between the cerebellar hemispheres on an axial view of the fetal head. The TCD is minimally affected by growth restriction and may therefore be more accurate in establishing gestational age. Sonographers should make two measurements of the TCD during late pregnancy scan.

The TCD is measured from the outer edge of the nearer cerebellar hemisphere to the outer edge of the more distant cerebellar hemisphere (OUTER TO OUTER). The following key steps should be followed in the measurement of the TCD:

A. After obtaining an adequate image for the BPD, rotate the transducer inferiorly and to image the cerebellum. The cerebellum will appear as peanut-shaped figure (or figure of 8) in the back of the skull behind and a little lower than the thalamus. Later in the third trimester, the cerebellum appears more pyramidal in shape.
B. Ensure good magnification of the image (with the fetal head occupying at least 50% of the image) and adjust the gain to obtain the sharpest image.
C. You will likely see the midline structures (thalamus, cavum septum pellucidum) but at times in the third trimester to optimize imaging of the cerebellum these shapes may be shadowed out. The head need not be imaged side to side on the screen. Especially late in gestation, the posterior fossa may need to be closer to the transducer to avoid shadowing from the skull over the cerebellum.
D. Ensure that the cerebellum is visible at the point of its greatest width. This requires scanning up and down the cerebellum to find the maximal width.
E. The calipers should be placed across the greatest width of the cerebellum. This will be perpendicular to the long axis of the skull. The top caliper should be placed touching the outer aspect of the nearer cerebellar hemisphere, and the lower caliper touching the outer aspect of the farther cerebellar hemisphere.
F. Reference images below.

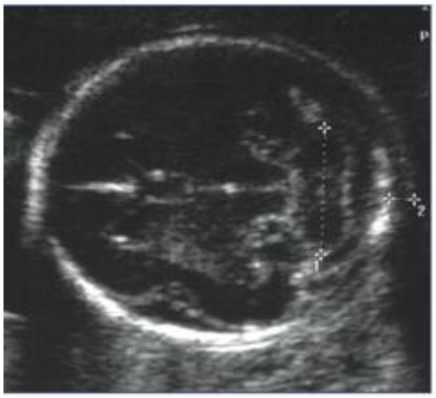

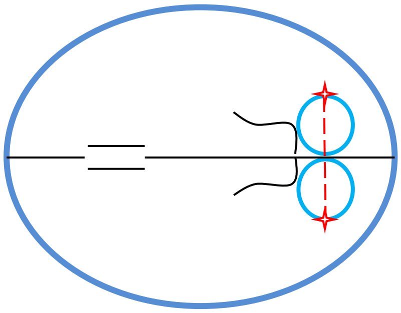

"Peanut" or "figure of 8"

REFERENCES

(1) Papageorghiou AT, Kennedy SH, Salomon LJ, et al. International standards for early fetal size and pregnancy dating based on ultrasound measurement of crown-rump length in the first trimester of pregnancy. Ultrasound Obstet Gynecol 2014; 44: 641–48.

(2) Chavez MR, Ananth CV, Smulian JC, Yeo L, Oyelese Y, Vintzileos AM. Fetal transcerebellar diameter measurement with particular emphasis in the third trimester: a reliable predictor of gestational age. Am J Obstet Gynecol 2004; 191: 979–84.

(3) Hadlock FP, Deter RL, Harrist RB, Park SK. Estimating fetal age: computer-assisted analysis of multiple fetal growth parameters. Radiology 1984; 152: 497–501.

(4) Papageorghiou AT, Kemp B, Stones W, et al. Ultrasound-based gestational-age estimation in late pregnancy. Ultrasound Obstet Gynecol 2016; 48: 719–26.
